# The need to fast-track a change in the nomenclature of the monkeypox virus

**DOI:** 10.4102/jphia.v16i1.1419

**Published:** 2025-09-03

**Authors:** Asukwo E. Onukak, Micah S. Otu, Juliet I. Mmerem

**Affiliations:** 1 Department of Internal Medicine, Faculty of Clinical Sciences, University of Uyo, Uyo, Nigeria; 2 Department of Internal Medicine, Dalhatu Araf Specialist Hospital, Nassarawa, Nigeria; 3 Department of Medicine, University of Nigeria Teaching Hospital, Ituku/Ozalla, Enugu, Nigeria

**Keywords:** monkeypox virus, mpox, International Classification of Diseases, global health, disease outbreaks

## Abstract

The nomenclature of monkeypox disease has been changed to mpox, and the clades of the causative virus have also been changed in keeping with international best practices. We commend the World Health Organization for its efforts in this regard while drawing attention to the need for a change in the name of the monkeypox virus. The continual use of the viral nomenclature can be considered stigmatising, discriminatory and inaccurate.

We seize this opportunity during the global outbreak of mpox to revisit the nomenclature of its causative virus. Applaudably, the World Health Organization (WHO) under the International Classification of Diseases and the WHO Family of International Health Related Classifications has changed the name of the disease from monkeypox to mpox.^1^ This is in line with its 2015 publication on WHO best practices for the naming of human infectious diseases,^2^ which pointed out that naming should be done in such a way as to minimise the unnecessary negative impact of disease names on trade, travel, tourism or animal warfare and avoid offending any cultural, social, national, regional, professional or ethnic groups. As laudable as the name change has been, it is necessary to promote its usage for wider acceptance as it is common to still see the older term being reflected in some literature. One possible reason affecting the application of the change in nomenclature is that the causative virus still shares the initial name of the disease prior to its modification.

We commend the efforts that have been made in modifying the nomenclature of the monkeypox virus variants via substituting the name of African regions previously attached to the clades by Roman numerals, giving rise to clades I and II.^1,3^ The International Committee on the Taxonomy of Viruses is responsible for the naming of viruses, and we recognise that there is already a process underway to consider renaming all orthopoxvirus species;^1^ however, more velocity is required as the delayed name change could negatively affect testing, contact tracing, vaccination and overall mpox response efforts by exacerbating the stigma surrounding the disease, given the elevated prevalence among sexual and gender minorities.^4^ A similar call has been made for the name change of Ebola virus disease and its causative virus, in which the different species of the *Orthoebolavirus* have been named after regional areas of first identification, thereby perpetuating regional stigmatisation without accurately reflecting changing virological characteristics of the disease.^5^ The non-offensive naming of severe acute respiratory syndrome coronavirus 2 (SARS-CoV-2) as the cause of coronavirus disease 2019 (COVID-19) is a notable recent example worth perpetuating, where geographical associations were avoided in the nomenclature of a novel virus and its attendant disease.^6,7^

Another reason why the change of name of the virus should be expedited is that rodents are believed to be its most likely reservoirs.^8^ Furthermore, case counts and epidemiological patterns also suggest that recent global mpox outbreaks are mainly sustained by human-to-human transmission.^8,9^ The recent evidence of recognised sexual transmission points to the virus becoming more efficient in its human-to-human spread.^8^ Hence, the continual use of this viral nomenclature, which has often been associated with racial slurs as illustrated by the use of photos of African patients in mainstream media to highlight the spread of the outbreak in the Global North, is not only inaccurate but also stigmatising and discriminatory.^9^
